# Plant-Based vs. Animal-Based Diets: Appetitive Traits and Dietary Patterns in Adults Based on Cross-Sectional Surveys

**DOI:** 10.3390/nu17030573

**Published:** 2025-02-04

**Authors:** Klaudia Wiśniewska, Katarzyna Małgorzata Okręglicka, Mariusz Jaworski, Aneta Nitsch-Osuch

**Affiliations:** 1 Department of Social Medicine and Public Health, Medical University of Warsaw, 02-091 Warsaw, Poland; katarzyna.okreglicka@wum.edu.pl (K.M.O.); aneta.nitsch-osuch@wum.edu.pl (A.N.-O.); 2 Doctoral School, Medical University of Warsaw, 02-091 Warsaw, Poland; 3 Department of Education and Research in Health Sciences, Medical University of Warsaw, 02-091 Warsaw, Poland; mariusz.jaworski@wum.edu.pl

**Keywords:** plant-based diet, appetite, eating, behaviour, weight, obesity, appetitive traits

## Abstract

Background: Dietary patterns play a crucial role in shaping eating behaviours and influencing health outcomes, such as body weight. Understanding how appetitive traits differ between plant-based and animal-based diets can provide insights into dietary strategies for weight management and improved health. Objectives: The aim of this study was to analyse the relationships between appetitive traits, as measured by the Adult Eating Behaviour Questionnaire (AEBQ), and dietary patterns in adults consuming plant-based or animal-based diets. It examined how these dietary patterns influence body mass index (BMI) and explored the differences in appetite-related traits between groups with different levels of plant and animal product consumption. Methods: A cross-sectional survey of 553 Polish adults was conducted using validated questionnaires, including the AEBQ and a food frequency questionnaire (FFQ). The participants were categorised into four dietary groups: high intake of both plant and animal products, low intake of both, plant-based diet, and animal-based diet. The data were analysed using SPSS version 14.0 software. Results: The participants on a plant-based diet had significantly lower BMIs and slower eating rates than those on an animal-based diet. Positive correlations were observed between ’food approach’ traits (e.g., food responsiveness, emotional overeating) and BMI, particularly in individuals with higher animal product consumption. Conversely, ’food avoidance’ traits (e.g., food fussiness, slowness in eating) were more prevalent among those on a plant-based diet. Conclusions: The results suggest that plant-based diets are associated with favourable appetitive traits and a lower BMI. These findings highlight the potential of plant-based diets to support weight control and improve eating behaviours. Further research is warranted to investigate the causal mechanisms underlying these associations.

## 1. Introduction

The popularity of plant-based diets (PBDs) is increasing steadily on a global scale [1,2]. A plant-based eating pattern is currently the most recommended dietary pattern by experts [3], due to its association with a reduced risk of various non-communicable chronic diseases, including cardiovascular disease [4,5,6] and obesity [7,8,9]. PBDs are also perceived as more ethical and environmentally sustainable [10,11]. There is no universally agreed upon definition of ’plant-based diets’ [12,13]. The term encompasses a multitude of dietary patterns, characterized by a reduction in the consumption of animal products and an increase in the intake of plant-based alternatives [14]. The reduction in or elimination of meat products, and in some cases fish, seafood, milk and dairy products, and eggs, is the primary aspect of this dietary pattern [3]. The proposed definition of PBDs, in this study, is “a dietary pattern that excludes foods of animal origin either completely or to a great extent” [12]. A well-balanced plant-based diet that focuses on whole, minimally processed foods has been shown to provide all essential nutrients and is linked to numerous health benefits [15,16]. Individuals following plant-based diets have been observed to have lower body weight [17] and improved body composition, enhanced glycaemic [18] and lipid metabolism [19,20,21], and lower blood pressure [8,22]. As a result, PBDs can effectively help prevent diseases, such as obesity, metabolic syndrome, and diabetes [23]. Although research has explored the general health benefits of plant-based diets, there is limited evidence directly comparing how plant-based and omnivorous diets influence key appetite traits, such as hunger and satiety responses, particularly in controlled settings. This is particularly important now, considering the significant changes in the market for plant-based foods in recent years and the rapid increase in the number of new plant-based products available [18,24,25,26,27]. Poland, along with France, the United Kingdom, Germany, Canada, and the United States, are actively introducing plant-based alternatives to meat and dairy products [24]. This initiative is reshaping the dietary patterns of individuals who restrict their meat consumption and adhere to a vegetarian or vegan diet, potentially influencing their appetite traits [26,27]. Consequently, it is pertinent to examine the variances in individual appetite traits between groups whose dietary preferences are centred on plant-based products and those based on animal-based products.

Understanding the connections between plant-based diets and specific appetite traits may be crucial in the context of efforts aimed at preventing an epidemic of overweight and obesity, especially since previous research has shown a correlation between appetitive traits and body weight [28]. At the individual level, weight gain is determined by the interplay of excessive food intake and reduced physical activity. These behaviours are influenced by both environmental factors and genetically predetermined appetite traits, which represent stable predispositions to food. The behavioural susceptibility theory of obesity postulates that individual differences in appetite traits are linked to an inclination to gain weight in response to a prevailing obesity-promoting environment [29]. So far, many questionnaires have been used to measure appetite traits. The most commonly used tools for adults are the Three Factor Eating Questionnaire (TFEQ) [30] and The Dutch Eating Behavior Questionnaire (DEBQ) [31], while for children, the most frequently used questionnaire is the Child Eating Behavior Questionnaire (CEBQ) [32]. The implementation of these questionnaires has facilitated a more comprehensive understanding of the individual appetite traits that contribute to an increased risk of weight gain or resistance to weight loss. These appetitive traits encompass food responsiveness, enjoyment of food, satiety responsiveness, emotional over- and undereating, food fussiness, and slowness in eating. The association between a plant-based diet and appetite traits, as assessed by the Adult Eating Behaviour Questionnaire (AEBQ), has not yet been empirically examined. The AEBQ questionnaire, validated as a reliable tool (Cronbach’s α > 0.70), imposes no supplementary costs for its administration [33]. Moreover, the questionnaire’s statements are lucid, and its administration is uncomplicated, as demonstrated by our previous studies in adults [34]. The AEBQ measures eight appetite traits: hunger (H), food responsiveness (FR), emotional overeating (EOE), enjoyment of food (EF), satiety responsiveness (SR), emotional undereating (EUE), food fussiness (FF), and slowness in eating (SE). The results show a positive correlation between a higher body mass index (BMI) and higher scores for the “food approach” (FR, EOE, and EF) and lower scores for the ‘food avoidance’ traits (SR, EUE, FF, and SE). The AEBQ questionnaire has been tested for its relationship with BMI in different populations, including Spanish and Mexican [33], Italian [35,36], Polish [36], Canadian [37], Chinese [38], Portuguese [39], and Australian [40].

An exploration of the appetite traits associated with a plant-based dietary pattern may yield insights into the observed correlation between plant-based consumption and reduced body weight. The available evidence indicates that a shift towards a plant-based diet may have a favourable impact on body weight and BMI in overweight and obese individuals [41,42,43,44]. The observed reduction in body weight can be attributed primarily to an increase in the consumption of dietary fibre, polyunsaturated fats and plant proteins, accompanied by a reduction in energy intake, saturated fats, and animal proteins [45,46]. The objective of this study was to ascertain the relationship between appetite traits and specific dietary patterns, such as plant-based diets and diets high in animal products, and to evaluate their impact on body mass index. This study uniquely emphasizes appetite traits (e.g., hunger, satiety, and emotional undereating) as mediating factors that connect dietary patterns to BMI. This perspective goes beyond the conventional focus on macronutrient composition or calorie intake. Additionally, by directly comparing PBDs with animal-based diets, this study offers valuable insights into how these distinct dietary patterns affect appetite regulation, something that has been understudied. This comparative approach may help bridge the gap between the physiological and behavioural aspects of eating.

## 2. Materials and Methods

A cross-sectional survey was conducted in compliance with the STROBE-nut Statement [47], and the authors present this study’s findings here. This study’s objectives were explained to each participant, and they were given the right to withdraw from this study at any time. Participation in the survey was voluntary for all individuals. This study was submitted to the Local Bioethics Committee of Warsaw Medical University for review and certification. The committee certified that this study complied with the principles of research ethics (AKBE/220/2024). All stages of this study were conducted following the Helsinki Declaration. Ethical considerations, such as confidentiality and anonymity, were observed in this study.

Initially, we examined the hypothesis, put forward by Hunot et al. [48], suggesting that individuals with obesity exhibit heightened scores for the ’food approach’ traits (FR, EOE, and EF) and diminished scores for the ’food avoidance’ traits (SR, EUE, and SE). Conversely, a reverse correlation is found among individuals with a normal BMI. The second hypothesis posits that adults consuming a diet rich in animal products would manifest elevated scores for the ’food attitude’ traits (FR, EOE, and EF) and reduced scores for the ’food avoidance’ traits (SR, EUE, and SE). On the contrary, individuals adhering to a plant-based dietary pattern would demonstrate an inverse relationship. The findings of this study will contribute to a more profound comprehension of the distinct appetite traits demonstrated by individuals adhering to plant-based and animal-based diets.

### 2.1. Participants and Recruitment

**Figure 1:**
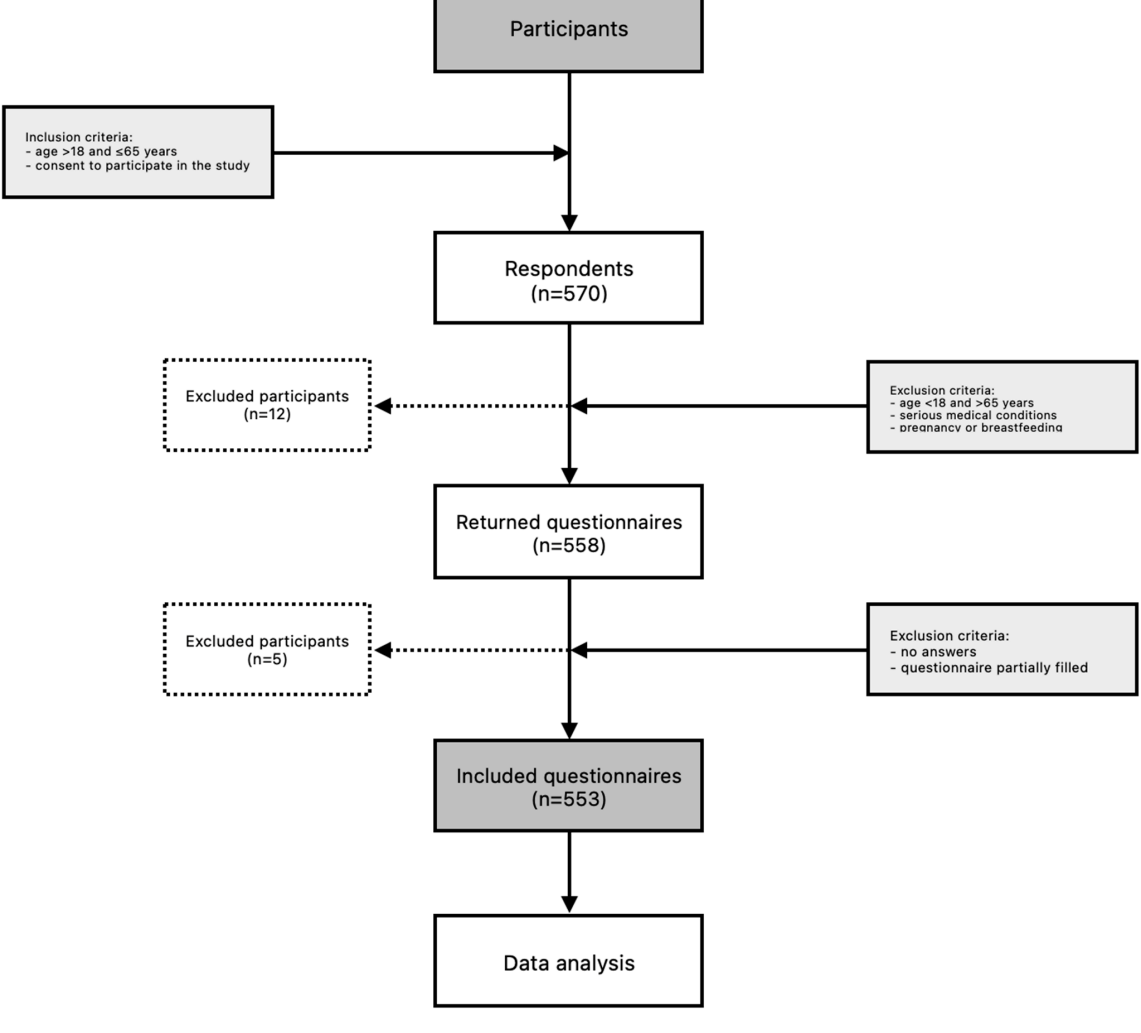
Flowchart: study design and data collection (n—number of participants).

The recruitment process for this study was conducted over six months, from November 2023 to April 2024. It was carried out electronically, leveraging advertisements on social media and specific health-related websites. Inclusion criteria encompassed individuals aged between 18 and 65 years who provided explicit consent to partake in this study. Individuals ineligible for participation in this study encompassed those below 18 or above 65 years of age, as well individuals with a severe medical condition, including alcoholism; major or extensive surgery; chronic kidney or liver disease; a history of myocardial infarction or unstable angina pectoris within the past six months; a history of stroke within the past six months; cancer within the past five years; a psychiatric condition that would hinder participation in this study; the use of enteral or parenteral nutrition; pregnancy or breastfeeding; and those who did not provide consent to participate in this study. The absence of disease was declarative in nature and was one of the questions included in the questionnaire. Following survey completion, respondents were invited to provide an email address in order to receive a complimentary e-book of recipes, with a market value of EUR 12. The initial cohort comprised 570 respondents who met the inclusion criteria. However, upon final analysis, the total number of participants included was 553. Initially, 12 participants were excluded from this study (7 women were pregnant or currently breastfeeding, and 5 indicated serious illnesses on the questionnaire that prevented participation, including having a stoma and undergoing oncological treatment). Subsequently, 5 participants were excluded due to incomplete completion of the questionnaire. Questionnaires that had not been fully completed were excluded from the analysis. A comprehensive overview of this study’s participants is depicted in Figure 1.

### 2.2. Data Collection

Data were obtained through the application of validated questionnaires tailored to the Polish population: Food Frequency Questionnaire (FFQ) and Adult Eating Behaviour Questionnaire (AEBQ) [48]. Furthermore, participants were asked to complete a Questionnaire for the Assessment of Dietary Habits, Lifestyle and Nutrition Knowledge (KomPAN) [49], which includes questions regarding demographic information, such as ethnicity, educational level, occupational status, and income. Additionally, weight and height data were collected to calculate BMI. In the initial phase of this study, participants were required to respond to a series of screening questions aimed at evaluating age, physiological status (pregnancy and breastfeeding), and prevailing medical conditions.

## 3. Results

### 3.1. Data Analysis

The statistical package for social sciences (SPSS) version 14.0 was used for the statistical analysis. During the initial phase of data analysis, we computed the descriptive statistics for the research indicators (Table 1). These indicators encompassed the dimensions derived from the eating behaviour questionnaire, the FFQ, and the outcomes obtained from the AEBQ scale. The statistical calculations involved determining the range (min-max), measures of central tendency (mean), dispersion (standard deviation), asymmetry, concentration (skewness, kurtosis), and tests of the normality of the distribution to ascertain the shapes of the obtained distributions. The Kolmogorov–Smirnov tests revealed that, in most cases, the variables exhibited statistically significant departures from a normal distribution, with the exception of the food avoidance index, which conformed to a normal distribution. Moreover, the variable measuring eating pleasure displayed a distinct concentration around the mean, indicating the leptokurticity of the distributions. Additionally, Cronbach’s alpha statistics were estimated to assess the reliability of the research tools for measuring the variables under investigation. A *p*-value ≤  0.05 was considered statistically significant.

### 3.2. Demographic Characteristics of Study Participants

This study included 553 participants, aged between 19 and 65 years (mean age = 35.90; SD = 9.28). The female respondents had a slightly higher average age than the male respondents, and had a lower average height, weight, and BMI. Further details are provided in Table 2.

Nearly four-fifths of the respondents possessed a university degree or higher, with the remaining individuals having predominantly completed secondary education. Notably, none of the participants had achieved only a primary or lower level of education. Additionally, over half of the respondents resided in urban areas with a population exceeding 100,000, while fewer than one-fifth hailed from rural or small-town settings. The majority of the respondents (approximately 45%) demonstrated a normal value for their BMI, while approximately one-third of them were classified as overweight, approximately one-fifth were obese, and less than 3% were underweight. Three-quarters of the respondents indicated permanent employment status, and one in ten reported engagement in educational pursuits. A summary of the basic demographic characteristics of the respondents is outlined in Table 3.

### 3.3. Factor Analysis of the FFQ: Animal Products and Plant Products

To reduce the research dimensions and determine the empirical structure of the FFQ questionnaire, an exploratory factor analysis using the principal components method (PCA) was conducted for 17 selected questionnaire items. The Kaiser–Mayer–Olkin (KMO) measure of sampling adequacy yielded a commendable result of 0.814, which is a good result [50,51]. Notably, the lowest single-item KMO measure stood at 0.698, a figure deemed as moderately acceptable (Field, 2009). Furthermore, Barlett’s sphericity test (*χ*^2^(136) = 3095.19; *p* < 0.001) indicated that the correlations between the items were high enough for analysis. The inter-item correlation matrix denominator was notably favourable at 0.003. The Haitovsky test, which produced a statistically non-significant result (*χ*^2^(136) = 1.64; *p* = 1.000), indicated that the item correlations did not exhibit excessive collinearity [48,49]. The Cattlela criterion indicated that two factors explained 41.74% of the variance. Subsequently, an orthogonal varimax rotation was selected to allocate the individual items to the extracted factors. The comprehensive findings of the factor analysis are detailed in Table 4.

To determine the measurement accuracy of the research tool used, Cronbach’s alpha statistics were calculated. The first scale, consisting of 10 items, demonstrated a high reliability, with a coefficient α = 0.823. Notably, the removal of any items would not result in an increased α value for the scale as a whole. This scale was designated as ‘Animal Products’, and a higher level corresponds to an increased consumption of these products by a respondent. The second scale, consisting of seven items, exhibited acceptable reliability, with a coefficient α = 0.693. Similar to the first scale, the removal of any items would not elevate the α value for the overall scale. This scale was labelled ‘Plant Products’, and a higher score indicates a greater consumption of plant products by the responder. The scales did not correlate with each other; therefore, they can be considered separable (rs = −0.009; *p* = 0.838).

### 3.4. Profiling of the Respondents

**Figure 2:**
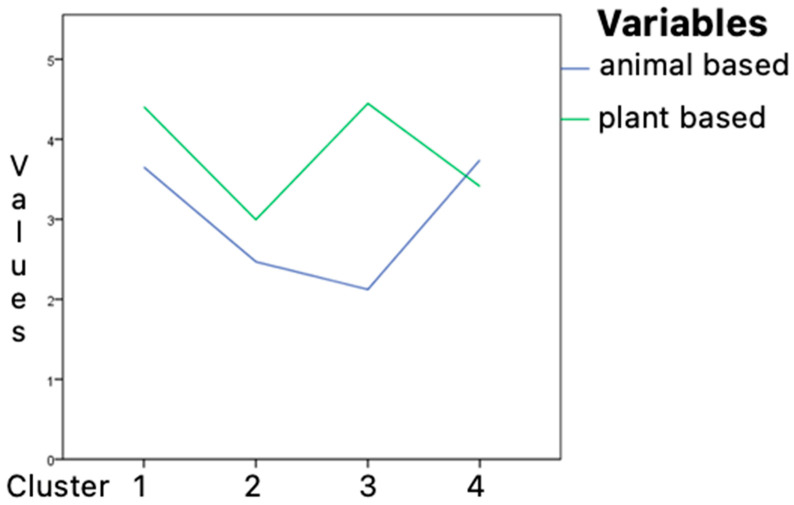
Final cluster centres of the four-cluster solution of the k-means analysis for variables obtained from the FFQ questionnaire.

**Figure 3:**
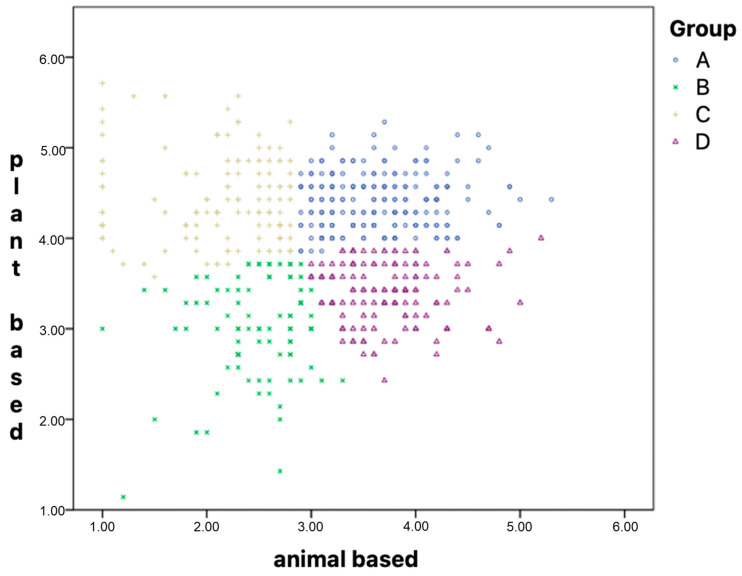
Scatterplot of variable-intensity scores obtained from the FFQ questionnaire against the four groups extracted by the k-means classification.

An effort was made to categorize the respondents into distinct groups (profiles) based on their consumption levels of both animal and plant products. This categorization was achieved through a k-means cluster analysis. This statistical analysis groups individuals based on shared characteristics, allowing for the identification of four unique dietary profiles that represent varying intensities of plant and animal product consumption. The findings of the analysis indicated that plant foods played a significantly more prominent role in the clustering process (F (3, 549) = 349.15; *p* < 0.001) compared to animal foods (F (1, 54) = 358.83; *p* < 0.001). Specifically, the impact of plant foods in forming the clusters was found to be statistically significant, while that of animal foods was comparatively less pronounced. Analysing the graphs of the final cluster centres (Figure 2) and the scatter plot for the pair of variables used to classify cluster membership (Figure 3), it was observed that the classification was based on segregating the scores into low and high combinations for both scales. This resulted in the identification of four distinct groups based on their consumption of plant and animal products [12].

The four groups were designated as (A) a high intake of animal and plant products, (B) a low intake of animal and plant products, (C) a plant-based diet, and (D) an animal-based diet. The allocation of the participants across these subgroups was not uniform (*χ*^2^ (3, N = 553) = 50.28; *p* < 0.001). Approximately one-third of the participants were assigned to the high-intake group (n = 203; 36.7%), while approximately one-fourth were allocated to the animal-based diet group (n = 136; 24.6%) and the plant-based diet group (n = 127; 23.0%). Less than one-fifth of the participants completed the questionnaire in a manner leading to assignment to the low-intake group (n = 87; 15.7%). A summary of the participants is detailed in Table 5.

### 3.5. Appetitive Traits and BMI

To test the hypothesis that adults with obesity exhibit higher scores on the ’food approach’ traits (FR, EOE, and EF) and lower scores on the ’food avoidance’ traits (SR, EUE, and SE), while an inverse relationship is anticipated for normal-weight individuals, intergroup comparisons were conducted using the Kruskal–Wallis H rank-sum test (Table 6) to examine this hypothesis.

Several differences were observed, and post hoc tests for the significant main effects were conducted using the Bonferroni–Dunn method. Hunger and food responsiveness were found to be significantly lower in individuals with an optimal BMI compared to those with obesity. It is noteworthy that the underweight group also exhibited a high median for these indicators; however, the size of the group and its internal variation within the present analysis suggest that this result should be considered as random. Similarly, the lack of statistically significant differences in the overweight group can be attributed to its internal variation.

This study found that emotional overeating increased significantly with higher BMI categories, indicating that the subjects with higher BMIs exhibited significantly higher levels of emotional overeating. Conversely, emotional undereating decreased significantly as the BMI category increased. It is important to note that the underweight and optimal BMI groups had statistically comparable levels of emotional undereating, which were lower than the comparable levels for the overweight and obese groups. In terms of food approach, the optimal BMI group had a significantly lower BMI than the overweight and obese groups, although the difference was not statistically significant. The small size of the underweight group and the inherent heterogeneity of the results prevented the differences compared with the other groups from being described as statistically significant. Food avoidance did not significantly differ between the underweight and optimal groups, but it was significantly lower in the underweight group than in the overweight and obese groups. The enjoyment of food, satiety responsiveness, and food fussiness did not differ between the BMI subgroups. In conclusion, the hypothesis was not fully confirmed.

### 3.6. Respondent Profiles and Body Weight and BMI

To examine the further hypothesis that those following a plant-based diet would have lower body weights and BMIs than those with a high meat intake, we conducted a series of intergroup comparisons using the Kruskal–Wallis H rank-sum test (Table 7).

A statistically significant difference was observed between the groups with regard to body weight (H(3) = 21.79; *p* < 0.001). The magnitude of the ε coefficient (ε^2^ = 0.04) suggests the presence of a very small effect. Subsequent post hoc tests, conducted using the Bonferroni–Dunn method, demonstrated that the individuals adhering to a plant-based diet exhibited a significantly (*p* = 0.006) lower weight compared to both the high-demand and low-demand groups (*p* = 0.012), and a lower weight than those following an animal-based diet (*p* < 0.001). No significant differences were observed between the three groups, indicating that the other diets exhibited comparable effects on body weights.

Additionally, a statistically significant difference was observed between the groups with regard to BMI (H(3) = 23.84; *p* < 0.001; ε^2^ = 0.04). The relationship was analogous to that observed for body weight. Those following a plant-based diet had a significantly (*p* = 0.002) lower BMI than those in the high-demand and low-demand groups (*p* = 0.046) or those following an animal-based diet (*p* < 0.001); however, there were no differences in the BMI between the three groups.

### 3.7. Respondent Profiles and Appetite Traits

To analyse the relationship between the consumption of animal and plant products and appetite traits, a correlation analysis was performed using Spearman’s non-parametric rho correlation test based on ranks (Table 8).

The findings suggest that as the consumption of animal products increased, the subjects’ food approach, hunger, and food responsiveness exhibited slight increases. Conversely, slowness in eating was observed to increase slightly with an increased intake of plant products, while food fussiness was found to decrease.

To test the hypothesis that adults with a high intake of animal products have higher scores for the traits of food approach (FR, EOE, and EF) and lower scores for the trait of food avoidance (SR, EUE, and SE), an inverse relationship was expected for the more plant-based eaters. To this end, a series of intergroup comparisons were conducted using the Kruskal–Wallis H rank-sum test. Two significant differences were identified, namely for the indexes of food fussiness (H(3) = 18.30; *p* < 0.001; ε^2^ = 0.03) and slowness in eating (H(3) = 16.51; *p* = 0.001; ε^2^ = 0.03). Nevertheless, in both instances, the effect size was found to be exceedingly modest.

The post hoc tests, calculated using the Bonferroni–Dunn method, demonstrated that the individuals with a high intake of plant-based products exhibited a significantly (*p* = 0.001) lower level of food fussiness compared to those with a low intake of plant-based products or those following an animal-based diet (*p* = 0.017). Additionally, the plant-based diet group demonstrated a reduced level of food fussiness (*p* = 0.049) in comparison to the low-intake group. In other words, the group with a low intake of plant products or the animal-based diet group exhibited a higher level of food fussiness than the group with a high intake of plant products or the plant-based diet group.

The group with low intake exhibited a significantly (*p* = 0.049) reduced incidence of ‘slowness in eating’ compared to those with a high intake and who followed a plant-based diet (*p* = 0.006). A similar observation was made in the case of the animal diet group (*p* = 0.013). In conclusion, the individuals with a high intake of plant products or a plant-based diet consumed their meals at a slower pace than those with a low intake of plant products or an animal-based diet. No other intergroup differences were identified. The hypothesis was not fully corroborated. Please refer to Table 9 for a comprehensive overview of all the data.

## 4. Discussion

This is the first study to quantify appetitive traits, as measured by the AEBQ questionnaire, in relation to the dietary intake of animal and plant products among adults, considering the BMI.

It aimed to determine whether appetitive traits correlate with the BMI, particularly if adults with obesity score higher on the ’food approach’ traits (FR, EOE, and EF) and lower on the ’food avoidance’ traits (SR, EUE, and SE), as observed by Hunot et al. [48]. Our results show that individuals with optimal BMI levels exhibit significantly lower levels of hunger and food responsiveness, while emotional overeating increases with a higher BMI. These observations are consistent with the psychological literature, which emphasizes the relationship between emotional overeating and weight gain. Eating can be used as a mechanism for coping with emotional tension and negative emotions (such as anger or anxiety), as well as a strategy for managing stress. Initially, such behaviours may lead to a subjective sense of relief and mood improvement; however, in the long term, individuals may fall into a vicious cycle of mood regulation through eating. Moreover, this behaviour can disrupt the physiological regulation of hunger, making it difficult to distinguish between physiological hunger and emotional hunger. Although numerous studies have highlighted the association between emotional eating and overweight/obesity, the underlying mechanisms of this relationship remain not fully understood and are difficult to definitively explain [50]. Our study suggests a potential complex interaction between appetite-related traits and the BMI index, warranting further investigation into the underlying mechanisms governing this association.

This research found a negative correlation between emotional undereating and BMI, with emotional undereating decreasing as BMI increases. The subjects with an optimal BMI had significantly lower food approach levels than those who were overweight or obese. For food avoidance, no difference was found between the underweight and optimal BMI participants, but both had lower levels than the overweight or obese groups. No significant differences were observed in the enjoyment of food, satiety responsiveness, or food fussiness across the BMI subgroups. It is noteworthy that the hypothesis was not entirely substantiated, aligning with findings from similar studies conducted in Australia [40] and Poland [36], and by Hana F. Zickgraf and Andrea Rigby. Only two AEBQ scales, emotional overeating and slowness in eating, were found to be associated with a baseline BMI in obese adults [52]. The differences observed in our study may be attributed to the disparate group selection. The participants in our study demonstrated a proclivity for utilizing social media profiles and health websites to monitor and manage their health. Additionally, there is also a possibility that the participants were cognizant of the correlation between dietary habits and body weight, which may have prompted some individuals to respond to the administered questionnaires in a manner that was perceived as socially desirable [29]. This phenomenon, known as socially desirable response bias, occurs when study participants modify their answers to align with social norms, the expectations of the researchers, or their own perception of what is socially acceptable. Such data distortion presents a challenge in research, as it can lead to the overreporting or underreporting of behaviours, ultimately affecting the reliability of the obtained results. This issue is particularly relevant in studies on dietary habits, body weight, and lifestyle, where the social pressure and expectations regarding healthy living may prompt respondents to portray themselves in a more positive light than is actually the case.

The absence of a correlation between BMI and the enjoyment of food and food fussiness may be attributed to the presence of diverse emotional eating disorders among the participants. It should be emphasized that emotional eating is not a binary phenomenon, but should rather be understood as a continuum of symptom severity, from mild to very intense forms. It is also crucial to consider both the causes and the degree of intensity of these behaviours. Psychological research indicates that emotional eating is associated with the consumption of specific types of food, which may vary individually; for one person, it could be a chocolate bar, while for another, ice cream. However, our study did not include questions regarding the type of food consumed in the context of coping with emotions, which constitutes a limitation. Future studies should take this factor into account to better understand the relationship between emotional eating and food preferences. The survey did not sufficiently inquire about psychological or familial issues that might influence food enjoyment and food selectivity.

Furthermore, the tendency to be fussy about food is not exclusively associated with BMI [52]. This indicates that individuals with different BMI values may exhibit similar levels of pickiness regarding food choices, suggesting that food selectivity is more related to individual taste preferences and eating habits rather than to the BMI itself. This highlights the complexity of this phenomenon. To better understand the results obtained, it is worth referring to the research in the field of cognitive psychology, which emphasizes the role of beliefs and thought processes in shaping behaviour. In the context of eating behaviours, an individual’s beliefs and thoughts may influence their pickiness through associations with prior experiences with specific food products, which in turn can shape their reactions and preferences toward certain types of food [53]. Satiety responsiveness may be contingent upon particular food selections or dietary patterns [54].

Moreover, the varying proportion of macronutrients in the respondents’ diets, particularly the proportion of protein, may have influenced their feelings of satiety, which was not assessed in this study. It is similarly probable that the respondents may have exhibited disparate perceptions and interpretations of feelings of satiety. Satiety is a highly subjective process influenced by various biological and psychological factors. A key aspect is that each individual assesses their state of satiety based on their previous eating experiences, which highlights individual differences in the perception of this state. From a scientific perspective, numerous mechanisms regulate satiety, including hormonal signals (e.g., leptin, ghrelin); neurotransmitters (e.g., dopamine); and sensory stimuli, such as taste, smell, and food texture. However, the final evaluation of satiety level often depends on subjective factors, such as expectations toward food, memories of previous meals, and emotional attitudes toward eating. Research shows that subjective evaluations of satiety can also be influenced by behavioural factors, such as portion size, the pace of eating, or the conditions under which a meal is consumed (e.g., the presence of others, distractions). As a result, individuals may respond differently to the same foods depending on the context. For example, the same meal may elicit different levels of satiety depending on whether it is consumed in a calm environment or a stressful situation. It is also important to note that individual interpretations of satiety are modulated by past experiences with specific foods; for instance, food that has previously been associated with a feeling of fullness may lead to a quicker attainment of satiety in the future [55,56].

It is also important to consider that adults may actively limit their energy intake to regulate their body weight, which may attenuate the impact of specific characteristics on their BMI. It would be beneficial for future research on the relationship between appetitive traits and BMI to consider these elements.

This investigation into the association between dietary habits and appetitive traits revealed a slight elevation in food approach, hunger, and food responsiveness levels among the individuals with a higher consumption of animal products.

This phenomenon may be ascribed to the heightened palatability of animal-based meals available on the market, which potentially stimulate appetite and increase food consumption more significantly than their plant-based counterparts [56]. Furthermore, our findings indicate a weak correlation between an increased consumption of plant products and slowness in eating. It is postulated that the prolonged slowness in eating may be attributed to the longer duration required for consuming plant products, and that the rate of eating is linked to the heightened energy intake and its potential impact on weight gain [57,58]. Additionally, while plant-based products are generally less processed, the market for plant-based products is rapidly evolving, with an increasing influx of highly processed offerings introduced by producers [24,25]. This factor could influence not only satiety but also the eating rate. Conversely, with the increased consumption of plant-based products, a decrease in selectivity regarding eating habits was observed. This shift may be attributed to individuals with PBDs being more open-minded, deriving heightened enjoyment from food, and consequently being more inclined to experiment with new culinary experiences [59]. The hypothesis that adults with a high intake of animal products exhibit higher scores on the ’food approach’ traits (FR, EOE, and EF) and lower scores on the ’food avoidance’ traits (SR, EUE, and SE) was not entirely substantiated. It was expected that individuals with a more plant-based diet would display the opposite pattern. The results demonstrate that individuals with a low consumption of plant-based products or an animal-based diet exhibited heightened food fussiness compared to those with a high intake of plant-based products or a plant-based diet. Additionally, it was observed that individuals with a high intake of plant-based products or a plant-based diet exhibited a slower eating pace than those with a low intake of plant-based products or an animal-based diet. First and foremost, plant-based diets are characterized by their high fibre content, which is found in large amounts in vegetables, fruits, legumes, and whole-grain products. Fibre not only requires longer chewing but also slows down the digestion process, making people who eat plants feel full for longer and eat more slowly. The scientific literature highlights that such foods require more effort to chew, which naturally affects the eating pace [60]. These observations align with the outcomes of the analysis of the relationship between eating habits and appetitive traits. Nevertheless, further investigation is warranted to address these complexities.

It is imperative to acknowledge that this study has its limitations. The small sample size, especially for the underweight group, reduces the ability to detect significant differences in appetite traits, increasing the risk of overlooking potential effects. Future studies should aim for larger and more balanced sample sizes to improve the statistical power, enhance the robustness of the findings, and ensure more reliable comparisons across the BMI categories. This study’s participants were not randomly selected; however, efforts were made to ensure sample diversity by disseminating information through various online channels. The completion of the questionnaires relied on voluntary participation, which may have introduced self-reporting bias, potentially resulting in an underestimation of the data and explaining the weak associations observed among the measured characteristics. This may have resulted in the introduction of sampling bias, as self-selection has the capacity to influence the results (e.g., individuals with a keen interest in health and diet may be over-represented) [61]. Furthermore, the data were obtained for the frequency of intake of individual products; however, accurate information on dietary nutrient intake was not collected, despite the known effect of dietary protein intake on satiety. Moreover, this study did not fully consider other lifestyle factors (e.g., physical activity levels, sleep patterns) that may influence BMI and appetite traits. It is therefore recommended that future research takes these factors into account. Additionally, the results may have been influenced by the specific population studied (Polish adults), and further research is required to generalise these results to other populations (e.g., different age groups or countries). Furthermore, our proposed classification of the participants into four groups is both intriguing and consistent with current definitions of plant-based diets. However, the unequal distribution of the participants between the groups may have had an impact on the data analysis. Moreover, the cross-sectional design of this study precludes the possibility of causal conclusions. It is recommended that studies, such as longitudinal studies or interventions, that could better explore the causal relationships between diet and appetite traits be conducted.

## 5. Conclusions

The findings of our study demonstrate a notable divergence in appetitive traits and body mass index between individuals adhering to plant-based diets and those who favour diets with a higher intake of animal products. The individuals following a plant-based diet exhibited lower BMI values, a greater tendency for slower eating, and lower food fussiness. These findings may be related to the higher fibre content and lower caloric density of plant-based foods. Plant-based diets, by promoting satiety, improving gut health, reducing reward-driven eating, stabilizing hunger hormones, and fostering mindful eating, can support appetite traits conducive to weight control. These mechanisms suggest that plant-based dietary patterns may be beneficial for long-term weight management and obesity prevention. In contrast, a diet high in animal products was found to correlate with a higher BMI and elevated scores for the “food approach” traits, such as food responsiveness and emotional overeating. These findings suggest that plant-based diets may positively influence appetite traits conducive to weight control. Plant-based diets can be a powerful tool in lifestyle medicine, addressing both disease prevention and overall well-being. Its role in reducing chronic disease risk, supporting mental health, and promoting sustainable nutrition makes it a cornerstone of holistic, evidence-based healthcare interventions [62]. However, the lack of a detailed analysis of the macronutrient intake in our study indicates the need for further research to better understand individual differences in dietary habits and their impact on appetite traits and body weight.

## Figures and Tables

**Table 1:** Descriptive statistics of the questionnaire survey indicators (n = 553).

	R	M	SD	Mdn	Sk	Kurt	D	α
FFQ								
Animal products	1.00–5.30	3.14	0.86	3.20	−0.41	−0.06	0.07 **	0.823
Plant products	1.14–5.71	3.95	0.71	4.00	−0.48	0.23	0.10 **	0.693
AEBQ								
Hunger	1.00–4.80	2.70	0.74	2.60	0.17	−0.10	0.07 **	0.653
Food responsiveness	1.00–5.00	2.87	0.81	2.75	0.23	0.08	0.10 **	0.677
Emotional overeating	1.00–5.00	3.17	1.07	3.20	−0.05	−0.83	0.07 **	0.885
Enjoyment of food	1.00–5.00	4.04	0.81	4.00	−0.84	1.04	0.12 **	0.859
Satiety responsiveness	1.00–4.75	2.28	0.68	2.25	0.40	0.34	0.10 **	0.590
Emotional undereating	1.00–5.00	2.41	0.94	2.40	0.23	−0.66	0.09 **	0.863
Food fussiness	1.00–4.80	1.91	0.78	1.80	0.95	0.71	0.12 **	0.836
Slowness in eating	1.00–5.00	2.46	1.00	2.25	0.43	−0.58	0.11 **	0.848
Food approach	1.00–4.76	3.11	0.66	3.12	−0.08	0.03	0.05 **	0.869
Food avoidance	1.00–4.44	2.25	0.54	2.22	0.29	0.18	0.04	0.813

R: range; M: mean; SD: standard deviation; Mdn: median; Sk: skewness of distribution; Kurt: kurtosis; D: Kolmogorov–Smirnov-test; α: α-Cronbach reliability measure; *p*: *p*-value; ** *p* < 0.01.

**Table 2:** Descriptive statistics of quantitative demographic indicators (N = 553).

	R	M	SD	Mdn	Sk	Kurt	D
Total							
Age	19.00–65.00	35.90	9.28	35.00	0.37	−0.35	0.07 **
Weight	45.00–136.00	75.53	17.32	73.00	0.80	0.50	0.08 **
Height	152.00–205.00	169.55	8.26	169.00	0.58	0.48	0.10 **
BMI	16.14–51.06	26.18	5.31	25.28	0.92	0.94	0.08 **
Women							
Age	19.00–61.00	36.07	9.36	35.00	0.34	−0.44	0.07 **
Weight	45.00–134.00	72.38	15.81	70.00	0.92	0.90	0.09 **
Height	152.00–188.00	167.24	6.56	167.00	0.28	0.18	0.08 **
BMI	16.14–51.06	25.85	5.41	24.77	1.04	1.22	0.10 **
Men							
Age	19.00–65.00	35.33	9.01	34.00	0.50	0.06	0.08
Weight	50.00–136.00	85.86	18.09	85.00	0.45	0.12	0.06
Height	155.00–205.00	177.12	8.73	178.00	−0.02	0.28	0.08 *
BMI	16.90–41.03	27.25	4.85	26.54	0.62	0.33	0.07

R: range; M: mean; SD: standard deviation; Mdn: median; Sk: skewness of distribution; Kurt: kurtosis; D: Kolmogorov–Smirnov-test; *p*: *p*-value, * *p* < 0.05 and ** *p* < 0.01.

**Table 3:** Summary of basic demographic characteristics of respondents (N = 553).

		N	%	*χ*^2^ Statistics
Gender	Female	424	76.7%	157.37 **
	Male	129	23.3%	
BMI	Underweight	12	2.2%	217.76 **
	Normal weight	248	44.8%	
	Overweight	178	32.2%	
	Obese	115	20.8%	
Professional status	Retirement or disability pension	2	0.4%	1185.76 **
	Parental leave, unemployment, etc.	29	5.2%	
	Studying	47	8.5%	
	Casual job	42	7.6%	
	Permanent employment	433	78.3%	
Education	Basic vocational	2	0.4%	936.64 **
	Primary education	3	0.5%	
	Secondary education	107	19.3%	
	Higher education	441	79.7%	
Accommodation	Village	84	15.2%	483.39 **
	Town with less than 20,000 inhabitants	36	6.5%	
	Town with 20,000 to 100,000 inhabitants	73	13.2%	
	City with more than 100,000 inhabitants	360	65.1%	

Note: the result of the analysis of the proportions of the numbers is reported as a chi-square test value with statistical significance in the form of ** *p* < 0.01.

**Table 4:** Empirical structure of the FFQ questionnaire: factor loadings and varimax rotation.

	Item	1	2
Q37	Dishes comprising white meat, such as chicken, turkey, and rabbit	0.784	−0.162
Q35	Cold cuts, sausages, or frankfurters	0.771	−0.242
Q36	Dishes made of so-called red meat, e.g., pork, beef, and veal	0.733	−0.253
Q33	Cottage cheese (including fromage frais and cottage desserts)	0.603	0.307
Q38	Fish	0.596	0.106
Q31	Milk (including flavoured milk, cocoa, and latte coffee)	0.578	0.051
Q34	Hard cheeses (including processed cheeses and moulded cheeses)	0.569	0.138
Q39	Eggs	0.556	0.216
Q32	Fermented dairy drinks, e.g., yoghurt, kefir (natural or flavoured)	0.530	0.436
Q28	Butter as an accompaniment to bread or food, for frying, baking, etc.	0.521	−0.084
Q43	Vegetables	−0.035	0.759
Q42	Fruits	0.062	0.709
Q25	Buckwheat, oatmeal, wholemeal pasta, or other coarse grains	−0.149	0.590
Q40	Dishes made from legumes, e.g., beans, peas, soy, and lentils	−0.401	0.563
Q53	Water, e.g., mineral water	0.169	0.470
Q23	Wholemeal bread	0.126	0.450
Q47	Canned vegetables or pickled vegetables	0.000	0.435
	Own values	4.22	2.88
	% of variance	24.81	16.93
	α	0.823	0.693

α: α-Cronbach.

**Table 5:** Summary of respondent profiles.

Group	Respondent Profiles	N	%	*χ* ^2^
A	high intake of animal and plant products group	203	36.7%	50.28 **
B	low intake of animal and plant products group	87	15.7%	
C	plant-based diet	127	23.0%	
D	animal-based diet	136	24.6%	

Note: the result of the analysis of the proportions of the numbers is reported as a chi-square test value, with statistical significance in the form of and ** *p* < 0.01.

**Table 6:** BMI categories and appetite traits.

	AEBQ	I: Underweight (n = 12)		II: Normal Weight (n = 248)		III: Overweight (n = 178)		IV: Obesity (n = 115)					
		Mdn	Mrang	Mdn	Mrang	Mdn	Mrang	Mdn	Mrang	H(3)	*p*	ε^2^	Post Hoc
A	Hunger	2.80	301.00	2.60	259.89	2.60	275.06	2.80	314.41	9.51	0.023	0.017	A.II < A.IV *
B	Food responsiveness	3.00	297.29	2.75	253.59	2.75	283.55	3.00	315.23	12.53	0.006	0.023	B.II < B.IV **
C	Emotional overeating	2.60	192.46	2.80	238.26	3.40	293.95	3.80	343.12	39.77	<0.001	0.072	C.I < C.IV * C.II < C.III ** C.II < C.IV ** C.III < C.IV
D	Enjoyment of food	4.33	288.13	4.00	291.94	4.00	269.95	4.00	254.54	4.98	0.173	0.009	n.s.
E	Satiety responsiveness	2.38	317.79	2.25	287.42	2.00	250.80	2.25	290.81	7.59	0.055	0.014	n.s.
F	Emotional undereating	3.20	397.83	2.60	303.75	2.20	258.18	2.00	235.83	24.08	<0.001	0.044	F.III < F.I * F.III < F.II * F.IV < F.I ** F.IV < F.II **
G	Food fussiness	1.30	202.33	1.80	278.75	1.80	276.01	1.80	282.56	2.83	0.419	0.005	n.s.
H	Slowness in eating	3.13	399.42	2.75	311.27	2.25	252.54	2.00	228.17	33.59	<0.001	0.061	H.III < H.I * H.III < H.II ** H.IV < H.I ** H.IV < H.II **
I	Food approach	3.09	254.67	2.94	245.33	3.18	289.10	3.35	328.90	23.16	<0.001	0.042	I.II < I.III * I.II < I.IV **
J	Food avoidance	2.61	372.21	2.33	308.57	2.14	248.13	2.17	243.66	24.79	<0.001	0.045	J.III < J.I J.III < J.II ** J.IV < J.I * J.IV < J.II **

* *p* < 0.05; ** *p* < 0.01; Mdn: median; Mrang: mean range; H(3): Kruskal–Wallis test; *p*: *p*-value; ε^2^: measurement of the strength of the effect by the epsilon2 coefficient; n.s., not significant.

**Table 7:** Respondent profiles and body mass and BMI.

	Group	Mdn	Mrang	H(3)	*p*	Post Hoc
	Body weight					
A.I	High-intake group	74.00	281.96	21.79 **	<0.001	A.III < A.I **
A.II	Low-intake group	75.00	291.39			A.III < A.II *
A.III	Plant-based diet	67.00	222.69			A.III < A.IV **
A.IV	Animal-based diet	77.50	311.12			
	BMI					
B.I	High-intake group	25.61	285.40	23.84 **	<0.001	B.III < B.I **
B.II	Low-intake group	25.28	280.21			B.III < B.II *
B.III	Plant-based diet	23.12	220.93			B.III < B.IV **
B.IV	Animal-based diet	26.39	314.76			

Mdn: median; Mrang: mean range; H(3): Kruskal–Wallis test, *p*: *p*-value, * *p* < 0.05; ** *p* < 0.01.

**Table 8:** Appetite traits and consumption of animal and plant products: Spearman’s rho rank correlation coefficients (N = 553).

AEBQ	Animal Products	Plant Products
Hunger	0.108 *	0.012
Food responsiveness	0.115 **	0.009
Emotional overeating	0.079	−0.001
Enjoyment of food	0.074	0.072
Satiety responsiveness	0.044	0.043
Emotional undereating	−0.001	0.023
Food fussiness	−0.022	−0.210 **
Slowness in eating	−0.048	0.204 **
Food approach	0.117 **	0.013
Food avoidance	−0.012	0.010

* *p* < 0.05; ** *p* < 0.01.

**Table 9:** Respondent profiles and appetite traits.

	AEBQ	I: High-Intake Group (n = 203)		II: Low-Intake Group (n = 87)		III: Plant-Based Diet (n = 127)		IV: Animal-Based Diet (n = 136)					
		Mdn	Mrang	Mdn	Mrang	Mdn	Mrang	Mdn	Mrang	H(3)	*p*	ε^2^	Post Hoc
A	Hunger	2.80	283.59	2.60	275.58	2.60	260.26	2.80	283.70	2.00	0.572	0.004	n.s.
B	Food responsiveness	2.75	284.17	2.75	274.19	2.75	264.91	2.75	279.38	1.21	0.751	0.002	n.s.
C	Emotional overeating	3.20	277.43	3.20	274.44	3.00	272.59	3.20	282.12	0.26	0.967	0.000	n.s.
D	Enjoyment of food	4.00	290.31	4.00	249.52	4.00	276.81	4.00	274.89	4.12	0.249	0.007	n.s.
E	Satiety responsiveness	2.25	280.49	2.00	261.31	2.25	272.77	2.25	285.78	1.46	0.693	0.003	n.s.
F	Emotional undereating	2.40	278.94	2.20	272.88	2.40	276.79	2.40	276.94	0.09	0.993	0.000	n.s.
G	Food fussiness	1.80	247.64	2.00	324.75	1.80	266.21	2.00	300.36	18.30	<0.001	0.033	G.I < G.II ** G.I < G.IV * G.III < G.II *
H	Slowness in eating	2.50	291.13	2.00	237.16	250	310.45	2.13	250.16	16.51	0.001	0.030	H.II < H.I * H.II < H.III ** H.IV < H.III *
I	Food approach	3.12	282.50	3.12	273.34	3.00	264.34	3.15	282.96	1.27	0.735	0.002	n.s.
J	Food avoidance	2.28	272.71	2.28	279.14	2.22	282.25	2.28	277.13	0.30	0.960	0.001	n.s.

* *p* < 0.05; ** *p* < 0.01; Mdn: median; Mrang: mean range; H(3): Kruskal–Wallis test; *p*: *p*-value; ε^2^: measurement of the strength of the effect by the epsilon2 coefficient; n.s. not significant.

## Data Availability

The data that support the findings of this study are available from the corresponding author [K.W.] upon request.

## Abbreviations

The following abbreviations are used in this manuscript:

MDPI	Multidisciplinary Digital Publishing Institute
DOAJ	Directory of open access journals
TLA	Three-letter acronym
LD	Linear dichroism
